# Gibberellin Biosynthetic Inhibitors Make Human Malaria Parasite *Plasmodium falciparum* Cells Swell and Rupture to Death

**DOI:** 10.1371/journal.pone.0032246

**Published:** 2012-03-07

**Authors:** Tomoko Toyama, Michiru Tahara, Kisaburo Nagamune, Kenji Arimitsu, Yoshio Hamashima, Nirianne M. Q. Palacpac, Hiroshi Kawaide, Toshihiro Horii, Kazuyuki Tanabe

**Affiliations:** 1 Laboratory of Malariology, Research Institute for Microbial Diseases, Osaka University, Yamadaoka, Suita, Osaka, Japan; 2 Department of Molecular Protozoology, Research Institute for Microbial Diseases, Osaka University, Yamadaoka, Suita, Osaka, Japan; 3 Department of Parasitology, National Institute of Infectious Diseases, Toyama, Shinjuku-ku, Tokyo, Japan; 4 Pharmaceutical Manufacturing Chemistry, Division of Medicinal Chemical Sciences, Kyoto Pharmaceutical University, Misasagi-Nakauchicho, Yamashinaku, Kyoto, Japan; 5 Institute of Agriculture, Tokyo University of Agriculture and Technology (TUAT), Saiwaicho, Fuchu, Tokyo, Japan; Institut national de la santé et de la recherche médicale - Institut Cochin, France

## Abstract

Malaria remains as one of the most devastating infectious disease, and continues to exact an enormous toll in medical cost and days of labor lost especially in the tropics. Effective malaria control and eventual eradication remain a huge challenge, with efficacious antimalarials as important intervention/management tool. Clearly new alternative drugs that are more affordable and with fewer side effects are desirable. After preliminary *in vitro* assays with plant growth regulators and inhibitors, here, we focus on biosynthetic inhibitors of gibberellin, a plant hormone with many important roles in plant growth, and show their inhibitory effect on the growth of both apicomplexa, *Plasmodium falciparum* and *Toxoplasma gondii*. Treatment of *P. falciparum* cultures with the gibberellin biosynthetic inhibitors resulted in marked morphological changes that can be reversed to a certain degree under hyperosmotic environment. These unique observations suggest that changes in the parasite membrane permeability may explain the pleiotropic effects observed within the intracellular parasites.

## Introduction

Malaria, caused by the genus *Plasmodium*, is one of the most common infections in the world responsible for about 1 million deaths per year. Artemisinin-based combination therapies (ACTs) are currently the first-line and only the best weapon against malaria [1]. Drug resistance, which rendered chloroquine and sulfadoxine-pyrimethamine infective for malaria control from 1970 to 1990s, remains as one of the greatest challenges facing malaria control today. Besides, ACT is 4 to 22× more expensive than common drugs and access is generally poor in African countries [2]. Also, although its clinical efficacy has not yet been compromised, there are recent reports that show the first evidence of artemisinin resistance [3]. Admittedly, the development of new inexpensive, effective and safe drugs with new mechanisms is strongly needed.

Plant-specific organelles and mechanisms in the phylum *Apicomplexa*, in which *Plasmodium* and other medically and veterinarily important pathogens are included, have been brought to focus as potential targets for new drugs since associated enzymes were found in plants and bacteria but not in animal metabolic pathways. Examples of these are plant-like vacuoles in parasite cells and the mevalonate-independent biosynthesis of isoprenoid in apicoplasts [4], [5]. The rationale was further strengthened with the demonstration that the apicoplast is essential for malaria parasite survival [6] and that metabolic pathways in the apicoplast are essential for parasite growth [7]. In addition, identification of inhibitors in these pathways might also result in synergistic drug combinations, which could have increased therapeutic value. The plant hormone abscisic acid (ABA) and ABA biosynthetic inhibitors have, likewise, been shown to affect parasite egress from infected host cells in *Toxoplasma gondii* [8].

In this study, we have preliminarily explored a wide variety of plant growth regulators, including plant hormones and corresponding inhibitors, to investigate their effect(s) on the growth of the most virulent human malaria parasite, *Plasmodium falciparum*. We have also used the apicomplexan parasite, *T. gondii* for comparison. *T. gondii* infects a broad spectrum of hosts and efficient drugs with low side effects and usable for human therapies are also highly needed. Plant growth inhibitors are commonly used in agriculture for years and have been synthesized in bulk, efficiently and cheaply, either naturally or artificially. Well-established manufacturing methods and facilities, as well as their safety profile (toxicity and teratogenicity) in animals, crops and humans are also available. Thus, plant growth inhibitors showing anti-apicomplexan activities might give valuable clues for prophylactic or therapeutic reagents effective for infectious diseases caused by protozoan parasites.

## Materials and Methods

### Chemicals

AMO-1618 (2-isopropyl-4-dimethylamino-5-methyl-phenyl-1-piperidinecarboxylate methyl chloride) was obtained from CALBIOCHEM (La Jolla, USA). FC-907 [*N,N,N,*-trimethyl-1-methyl-(2′,6′,6′-trimethylcyclohex-2′-en-1′-yl)prop-2-enylammonium iodide] was kindly provided by Prof. Y. Kamiya (RIKEN, Japan). LysoTracker® Red DND-99 was obtained from Invitrogen (San Diego, USA). All other chemicals were purchased from Wako Pure Chemicals (Osaka, Japan).

Prior to use and dilution at various concentrations, AMO-1618, chlorocholine chloride, prohexadione and FC-907 were dissolved in distilled water. INA and ancymidol were dissolved in DMSO and diluted in 0.1% DMSO. Paclobutrazol and uniconazole P were dissolved in ethanol and diluted in the culture medium.

Enantiomeric resolution of racemic INA (Wako) was carried out by high performance liquid chromatography (HPLC; LC-908, Japan Analytical Industry Co., Ltd, Japan). Elution order of enantiomers was determined by an ultraviolet absorption detector (SPD-10A, Shimadzu, Japan) using Chiralcel OD column [25×0.46 cm, Daicel Chemical Industries, Japan, mobile phase: n-hexane-2-propanol (7∶3, v/v), flow rate: 1.0 ml/min]. All resolutions were carried out at 26°C; detection at 275 nm. The assignment of the peaks was achieved by comparison with the synthesized (*S*)-form INA sample as previously described [9]: peak with shorter retention time correspond to the (*R*)-form and the peak with longer retention time to the (*S*)-form. Percentage purity of the products was calculated to be 97.0% and 99.5% for (*S*)- and (*R*)-forms, respectively, based on peak areas.

### In vitro culture

*P. falciparum* strain 3D7 was cultured at 3% hematocrit in RPMI 1640 supplemented with 10% human serum, 50 mg/l hypoxanthine and 25 mg/l gentamicin, as previously described [10]. Cultures were maintained at 37°C in a gas mixture of 5% CO_2_, 5% O_2_, and 90% N_2_.

The *T. gondii* strain 2F tachyzoites, derived from strain RH, constitutively expressing cytoplasmic β-galactosidase (β-gal), were routinely grown in Vero cells (African green monkey kidney, strain ATCC CCL-81™) at 37°C under 5% CO_2_ in RPMI 1640 medium containing 10% fetal calf serum [11].

### In vitro antimalarial assay of plant growth regulators

Asynchronous *P. falciparum* 3D7 was used. Various concentrations of compounds in appropriate solvents (water, ethanol or DMSO) were prepared and added to 12-well plates. Starting parasitemia was at 0.1% in 2.5 ml culture medium. Growth was assessed after 72 h by percentage parasitemia using thin blood smears. The number of parasitized erythrocytes over a total of 3,000 erythrocytes was examined. Drug-free control cultures were run simultaneously.

For *T. gondii* studies, confluent Vero cell cultures were incubated for 2 days and infected with 2.5×10^5^ tachyzoites in RPMI 1640 medium containing 3% FCS using a 96-well plate. Tachyzoites were harvested after 2 days and β-gal activity was analyzed using a colorimetric assay [12].

### Morphological effects of gibberellin biosynthetic inhibitors on P. falciparum

Tightly synchronized parasites within 4 h life span were prepared using 5% sorbitol treatment and percoll centrifugation. Synchronized parasites were treated with either 50 µM INA or 250 µM AMO-1618 from 0 h (ring), 20 h (immature trophozoite), 28 h (mature trophozoite) or 36 h (schizont). Giemsa-stained thin-blood smears were prepared after 4, 8 and 12 h treatment. Digital imaging was performed on a HC-300 (Fujifilm, Japan) and representative parasite images are shown.

### Fluorescence Microscopy

Thin-blood smears of infected erythrocytes treated with INA were stained with acridine orange (100 µg/ml). Fluorescence microscopy and confocal imaging were carried out using the Axioplan 2 microscope (Zeiss, German) and SPOT PS-BW CCD camera (Seki Technotron Corp., Japan). Filter sets for green fluorescence (green: nucleoli; emission LP515, excitation BP 450–490) and red fluorescence (red: cytoplasm; emission LP590, excitation 546/12) were used. Nile Red staining was carried out by addition of 1 µg/ml dye to the culture medium 1 h prior to microscopic analysis. Nile Red was excited at 546 nm and emission detected above 590 nm. To stain with rhodamine 123 and LysoTracker® Red DND-99, 10 ng/ml rhodamine or 75 nM LysoTracker were added to the culture and incubated for 1 h. Confocal images were obtained with excitation above 546 nm and emission above 590 nm.

### Electron microscopy

Intraerythrocytic parasites treated with 50 µM INA for 6 h, 250 µM AMO-1618 for 8 h or 0.1% DMSO for 6 h were centrifuged at 3500× g for 1 min and fixed in 2% glutaraldehyde in 1× PBS for 1 h. After washing in 1× PBS, fixed cells were treated as described [13]. Sections were viewed in a transmission electron microscope JEM-1011 (JEOL Ltd., Japan).

### Effects of osmotic pressure on INA-treated P. falciparum

*P. falciparum* in RPMI 1640 containing 10% human serum was grown to 5–10% parasitemia and synchronized by sorbitol treatment. After 21 h, 0.9 ml of early trophozoite-stage parasites was mixed with 0.1 ml of 1×, 2×, 3× and 4× PBS and treated simultaneously with 50 µM INA or 0.1% DMSO for 8 h (INA stock solution was prepared as 50 mM in DMSO and diluted to 1/1000 for assay). Parasitemia was determined using Giemsa-stained thin smears by a laboratory technician blinded to treatment assignments. Each treatment was replicated thrice and experiments were performed twice.

Volumes of 0, 0.1, 0.2 and 0.4 ml sterilized water was adjusted to 0.4 ml by mixing with appropriate amounts of RPMI 1640 medium containing 10% human serum for the different dilution series. Hyposmotic media were mixed with 0.6 ml of *P. falciparum* culture synchronized to early-stage trophozoites.

### GC-MS analysis of sterols in P. falciparum

Parasites were cultured in 5 ml RPMI 1640 supplemented with 1% serum free AlbuMAX® II (Invitrogen). Parasitized erythrocytes were collected by centrifugation and hemolyzed in 0.54 ml of 0.15% saponin in RPMI 1640. Erythrocyte-free parasites were washed twice with 4 ml of 1× PBS and suspended in 0.4 ml of 1× PBS. Geranylgeraniol at 50 µg was added to the suspension as an internal standard and extraction with 1 ml of chloroform was carried out 3 times by shaking the mixture at 37°C.

Chloroform extracts were pooled and 1 µl solution was directly injected into the GC-MS (gas chromatography/mass spectroscopy) instrument (JEOL; JMS-Bu25 GCMate: ionization energy at 70 eV, filament current at 300 mA) with a DB-5 capillary column [0.25 mm i.d.×15 m, film thickness of 0.25 mm (J&W Scientific, USA)]. The He carrier gas flow rate was 1 ml/min (constant flow), the injector temperature was 250°C, and the samples were introduced by splitless injection. After injection, the oven temperature program was held at 80°C for 1 min, then increased to 200°C at 30°C min^−1^, followed by a further increase to 300°C at 15°C min^−1^ after which it was held constant at 300°C for 2 min.

For quantitative analysis, commercially available cholesterol and geranylgeraniol (Sigma) was detected by GC-MS and corresponding peaks were used as authentic standards for cholesterol and geranylgeraniol, respectively. Cholesterol was calculated based on ratio of peak areas with geranylgeraniol.

## Results

### Antimalarial activity of gibberellin biosynthetic inhibitors

**Figure 1:**
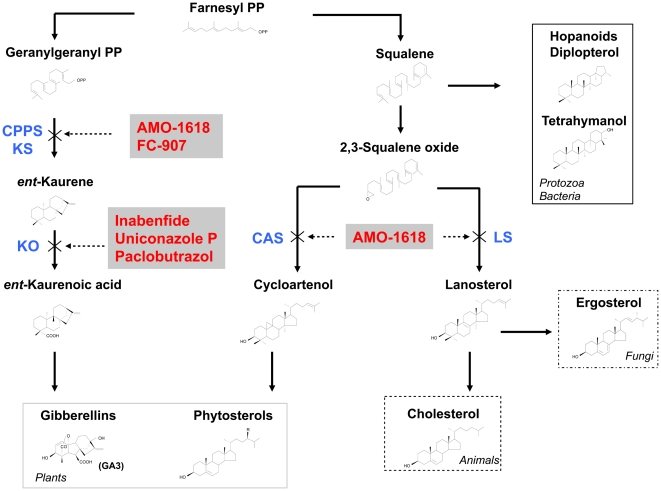
Isoprenoid biosynthetic pathways and known inhibitors in various organisms. Broken arrows indicate blocks in the biosynthesis due to specific inhibitors. “R” indicates various functional groups specific to individual compounds. CPPS, copalyl-diphosphate synthase (EC 5.5.1.13); KO, *ent*-kaurene oxidase (EC 1.14.13.78); CAS, cycloartenol synthase (EC 5.4.99.8); LS, lanosterol synthase (EC 5.4.99.7); KS, *ent*-kaurene synthase (EC 4.2.3.19); PP, pyrophosphate.

All inhibitors of gibberellin used in this study affect synthesis of gibberellin in plants (Fig. 1). Generally, the inhibitors blocked *P. falciparum* growth *in vitro* (Table 1, Fig. S1); although, results were admittedly different among the inhibitors as well as between *Plasmodium* and *Toxoplasma*. For example, inabenfide (INA) exerted strong activities in both *P. falciparum* and *T. gondii*, whereas prohexadione exhibited no cytotoxicity to *P. falciparum* even at 500 µM. Likewise, ED_50_s of some inhibitors varied greatly between *P. falciparum* and *T. gondii*, such as AMO-1618: 10.3±2.22 µM in *P. falciparum* and >1000 µM in *T. gondii*. Apparent difference in effective inhibitor concentrations between the two parasite species could be species-specific barrier on membranes for drug uptake or inhibitory mechanisms in *P. falciparum* and *T. gondii* [14].

**Table 1:** ED_50_ of the gibberellin biosynthetic inhibitors.

Reagents	ED_50_ of *P. falciparum* (µM)	ED_50_ of *T. gondii* (µM)
Chlorocholine Chloride	8.94±26.08	>10000
AMO-1618	10.3±2.22	>1000
FC-907	4.26±0.14	2206.7±2.46
Inabenfide	2.94±0.15	17.0±1.32
Paclobutrazol	27.1±2.69	120.6±5.38
Uniconazole P	30.7±6.20	97.4±8.66
Ancymidol	322.9±13.1	>1000
Prohexadione	>500	>10000
(*S*)-Inabenfide	2.78±0.51	24.04±1.07
(*R*)-Inabenfide	6.32±0.81	6.23±1.65

Growth inhibitory effects of gibberellin biosynthetic inhibitors and enantiomers of INA to *P. falciparum* and *T. gondii* are shown. Values are the mean ± standard deviations (SD) from three independent experiments, with each treatment duplicated twice. N.D.; not determined.

Some of the tested inhibitors have an asymmetric carbon, and their enantiomers are known to differ significantly in their biological properties [15]. (*S*)-forms are biologically more active than (*R*)-forms in higher plants [16]. In order to examine the chirality effect of these inhibitors, we separated enantiomers from commercially available INA by HPLC using a chiral column (Fig. S2) since marketed INA is racemic. The (*S*)-form was slightly more potent than the (*R*)-form, as demonstrated by ED_50_ values of 2.78±0.51 µM and 6.32±0.81 µM, respectively in *P. falciparum* (Table 1). Notably, the (*R*)-form was more effective in inhibiting growth of *T. gondii*.

### Morphological changes induced by gibberellin biosynthetic inhibitors

**Figure 2:**
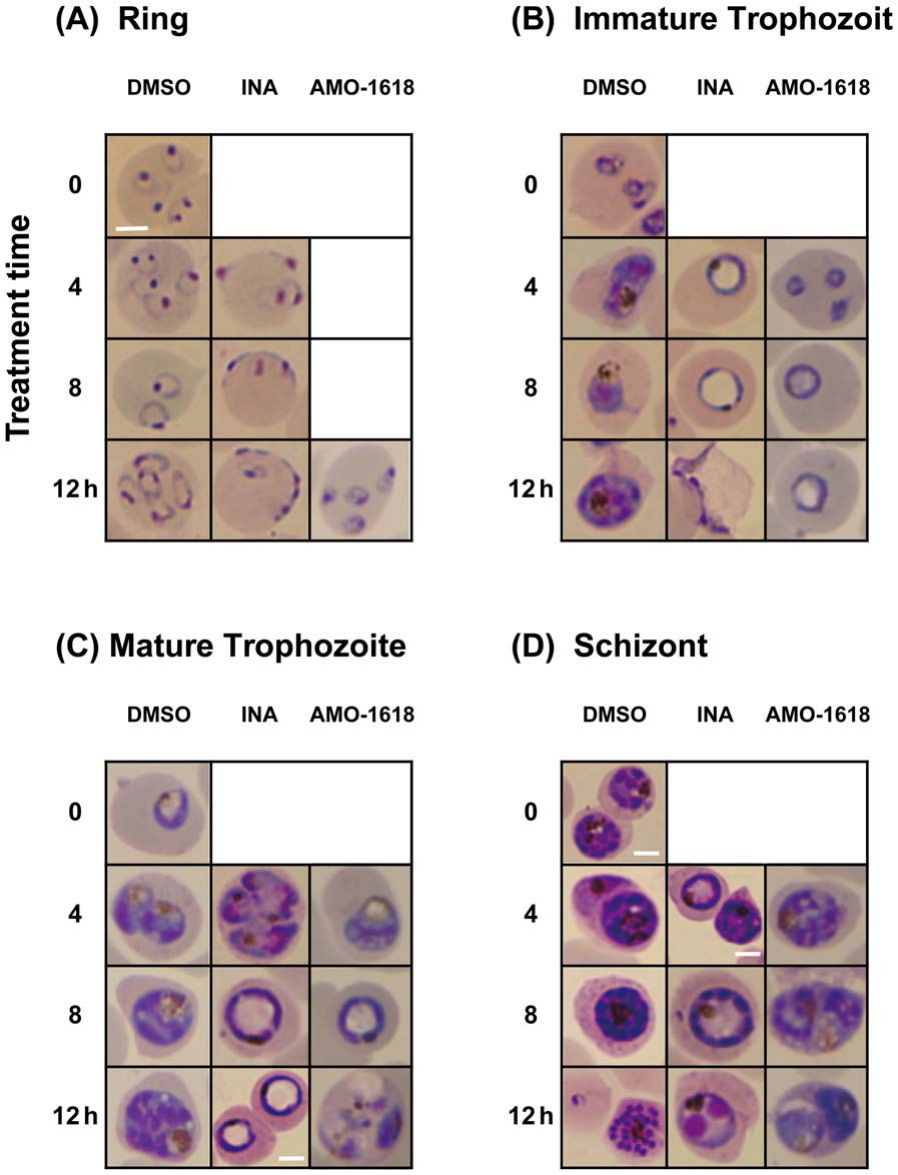
Effect of INA and AMO-1618 on intraerythrocytic development of *P. falciparum*. Tightly synchronized parasite cells that have undergone 5% sorbitol treatment and percoll density gradient centrifugation (window period: 4 h) were treated with 1 µl/ml DMSO, 50 µM INA or 250 µM AMO-1618 at different parasite stages: after 0 h (ring, A), 20 h (immature trophozoite, B), 28 h (mature trophozoite, C) and 36 h (schizont, D). Cultures were examined after 4, 8 and 12 h using Giemsa thin blood smears. Scale bar: 3 µm; all images without a scale bar are displayed at the same scale as the left uppermost image in (A).

We also evaluated morphological and physiological effects induced by the gibberellin biosynthetic inhibitors. In what appears to be the center of the parasite, a Giemsa-unstained region was prominent within 4 h after treatment with INA (Fig. 2 B). The “haloed” parasites swelled and ruptured within 12 h of culture in immature trophozoite-stage. Rings were seldom found when early trophozoite-stage parasites were treated with INA. When INA was applied at late stages of the parasite, *i.e.* in mature trophozoites and schizonts, a similar swelling was also observed although the stained periphery appears broader with merozoites bloated in appearance and few in number (Fig. 2 C, D). Ring-stage treated parasite cultures were usually appliqué forms (rings appearing on the periphery of the erythrocytes) (Fig. 2 A).

Another type of gibberellin biosynthetic inhibitors, AMO-1618 and FC-907, which prevent cyclization reaction in the early steps of gibberellin biosynthesis (Fig. 1) [15], [17], also caused parasite cells to swell. Swelled parasites were found within 8 h and 24 h after AMO-1618 treatment (Fig. 2, S3), but the altered morphology on schizonts was different: parasites appear abnormally disordered inside erythrocytes (Fig. 2 C, D). A similar morphological change in *Plasmodium* cultures were observed when greater than 2 µM INA were used (data not shown). Notably, both INA and AMO-1618 did not appear to damage erythrocytes, because *P. falciparum* could infect erythrocytes that had been treated for 24 h with 50 µM INA or 250 µM AMO-1618 without any significant inhibitory effects (Fig. S4).

At 2 µM, INA-induced morphological changes were microscopically visible at 24 h in asynchronous parasite cultures. In order to determine if INA and AMO-1618 targets a specific stage during intraerythrocytic development, we increased concentrations of INA to 50 µM and AMO-1618 to 250 µM. Both inhibitors at these concentrations provoked morphological changes more rapidly than concentrations at their ED_50_s (Fig. S5). The use of increased concentrations of inhibitors also allowed us to measure the intrinsic sensitivity of the parasites *in vitro*, and provided clues to the maximal response that can be produced by these inhibitors, as well as their toxicity to the human erythrocytes. Even after 24 h incubation, use of 0.1% DMSO alone gave no effects on growth and morphology of malaria parasites. Parasite swelling and rupture was also observed on *P. falciparum* cultures treated with other gibberellin biosynthetic inhibitors, such as paclobutrazol and uniconazole P (Fig. S5). These inhibitors, as well as INA, are known to block cytochrome P450-dependent monooxygenases (CYP701A) in plants [15]. Interestingly, the time and the concentrations necessary to provoke morphological changes were different among these compounds, presumably due to differences in solubility and lipophilicity.

### The ‘haloed’ trophozoites

**Figure 3:**
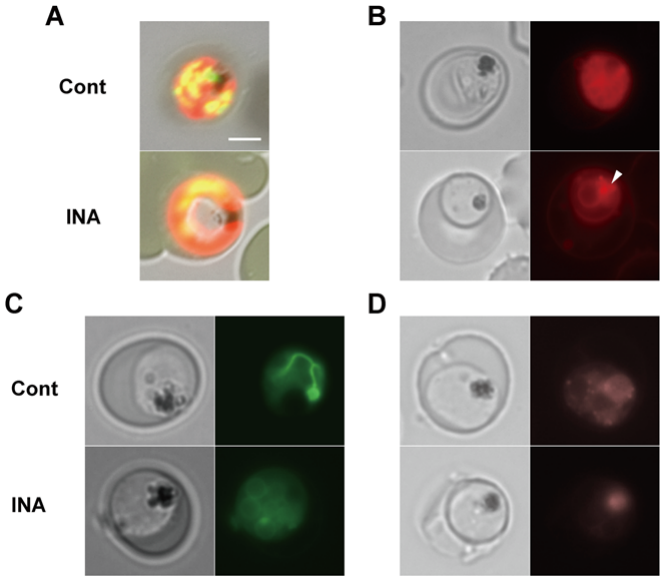
Fluorescence microscopy of INA-treated parasites. Infected erythrocytes were stained with (A) acridine orange, (B) Nile Red, (C) rhodamine 123 and (D) LysoTracker™ Red DND-99. INA was introduced at: (A) 100 µM for 9 h, (B) 50 µM for 6 h, and (C and D) 50 µM for 4 h. 100 µg/ml acridine orange was applied to thin blood smears made from intraerythrocytic parasites treated with INA. For other fluorescence dyes, *P. falciparum* cultures were incubated with probes for 1 h at the following concentrations: Nile Red, 1 µg/ml; rhodamine 123, 10 ng/ml; and LysoTracker® Red DND-99, 75 nM. Cells were not washed prior to fluorescence microscopy to minimize damage due to osmotic changes. Scale bar, 3 µm. An arrow indicates a lipid body in (B).

The “haloed” parasites were stained with various fluorescent probes to visualize intracellular components. The center of the trophozoites was not stained with acridine orange (Fig. 3 A). Both nuclei and cytoplasm appear to be on the periphery, as seen in the Giemsa-stained images. Nile Red, used to visualize various lipid-rich compartments such as membranes, endoplasmic reticulum (ER) and lipid bodies [18], revealed a simpler staining pattern surrounding what appears to be fewer merozoites in contrast to the control. Merozoites also appear bloated with intense spots of fluorescence (presumed as lipid bodies, Fig. 3 B). In normal mature stage trophozoites, rhodamine 123 showed thread-like branched mitochondria, characteristic of the mitochondrial development at the later stages where each daughter merozoite receives a branch of the parent organelle (Fig. 3 C) [19]. In INA-treated parasites, however, branched mitochondria were rarely seen. Although fluorescence intensity in the mitochondria started to decrease after 2 h, parasites still have significant fluorescence over the entire parasite cytosol, except in the food vacuole, similar to untreated parasite cells. This observation suggest a membrane potential generated by the parasite's outer plasma membrane [20], [21] and that INA effect likely induced a specific reduction in the mitochondrial membrane potential alone.

These differences in the staining patterns were not evident when ring-stage parasites were observed (data not shown), which suggest that various membranes were remarkably affected by INA only when parasites reached the trophozoite stage. The timing is coincident at a time when the parasite starts to actively generate membranes needed for growth and schizogony [22].

**Figure 4:**
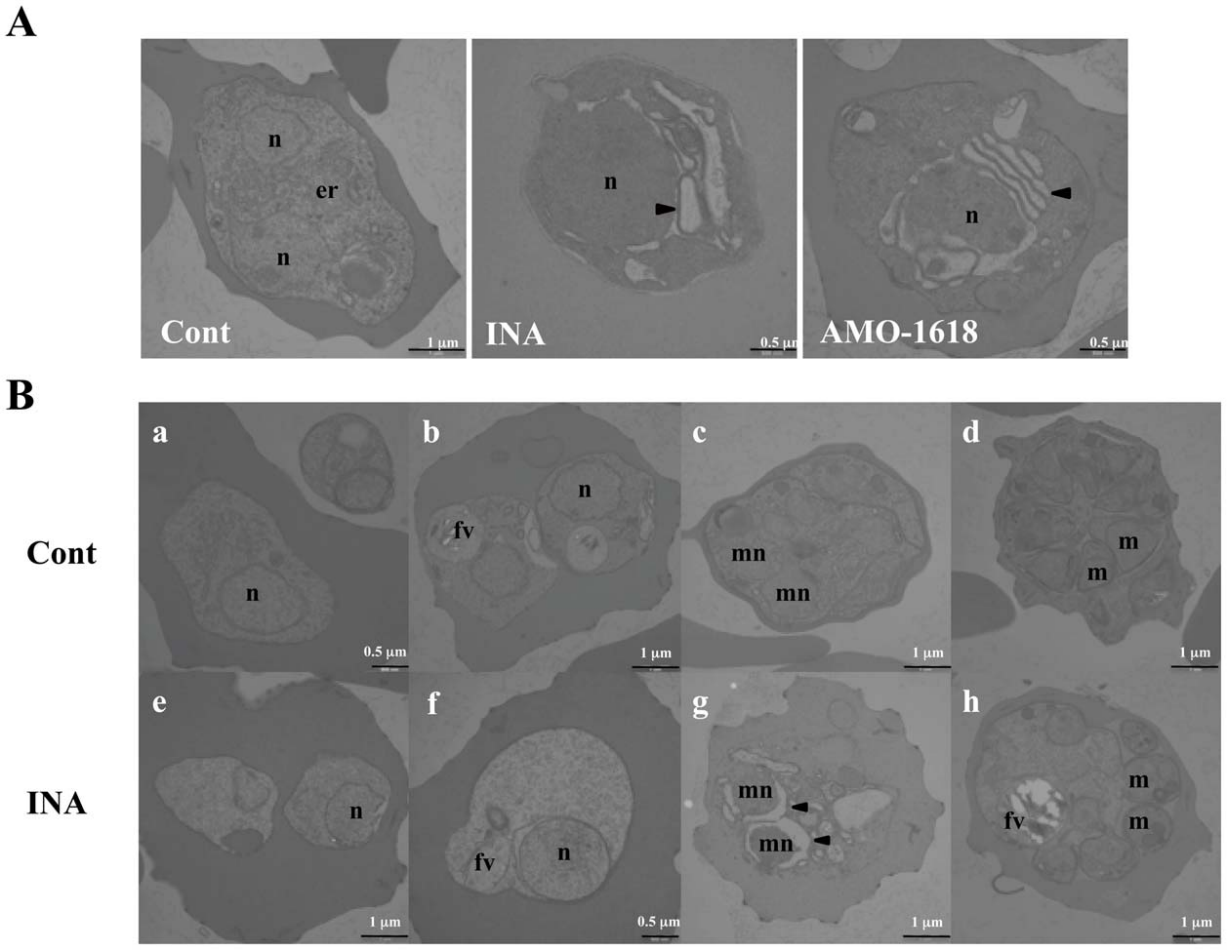
Transmission electron microscopy of parasitized erythrocytes treated with inhibitors. (A) Sections through an erythrocyte containing a trophozoite-stage parasite exposed to 0.1% DMSO for 6 h, 50 µM INA for 6 h or 250 µM AMO-1618 for 8 h, respectively. (B) Asynchronized parasites were treated with 0.1% DMSO (a–d) or 50 µM INA for 6 h (e–h). Sections from representative stages during intraerythrocytic development: ring- (a and e), early trophozoite- (b and f), mature trophozoite- (c and g) and schizont-stage parasites (d and h) are shown. Nuclei (n), food vacuoles (fv), merozoites (m), nuclei of merozoites (mn) and abnormal gaps between the nuclei and the nuclear envelopes (arrowheads) are indicated. Scale bar is indicated at the bottom of the images.

Electron microscopy also revealed fine intracellular differences at the trophozoite and schizont-stage parasite (Fig. 4 A, B). When trophozoites were treated with INA or AMO-1618 (Fig. 4 A), membranes of the nuclear envelope and ER were thicker and intensely stained compared to control parasites. Conspicuously unfamiliar spaces appeared around the ER and daughter nuclei (Fig. 4 A, INA and AMO-1618 panel). Using asynchronous cultures, these spaces were more readily observed at mature-stage parasites than early trophozoites (Fig. 4 B, b and f vs. c and d). No differences were evident in the plasma membranes of both host and parasite cells during the intraerythrocytic life cycle (Fig. 4 B). These results suggest that the gibberellin biosynthetic inhibitors appear to affect components of various intracellular membranes of the parasites, and the effects were especially evident during schizogony.

### INA effect and osmotic pressure in parasite cells

**Figure 5:**
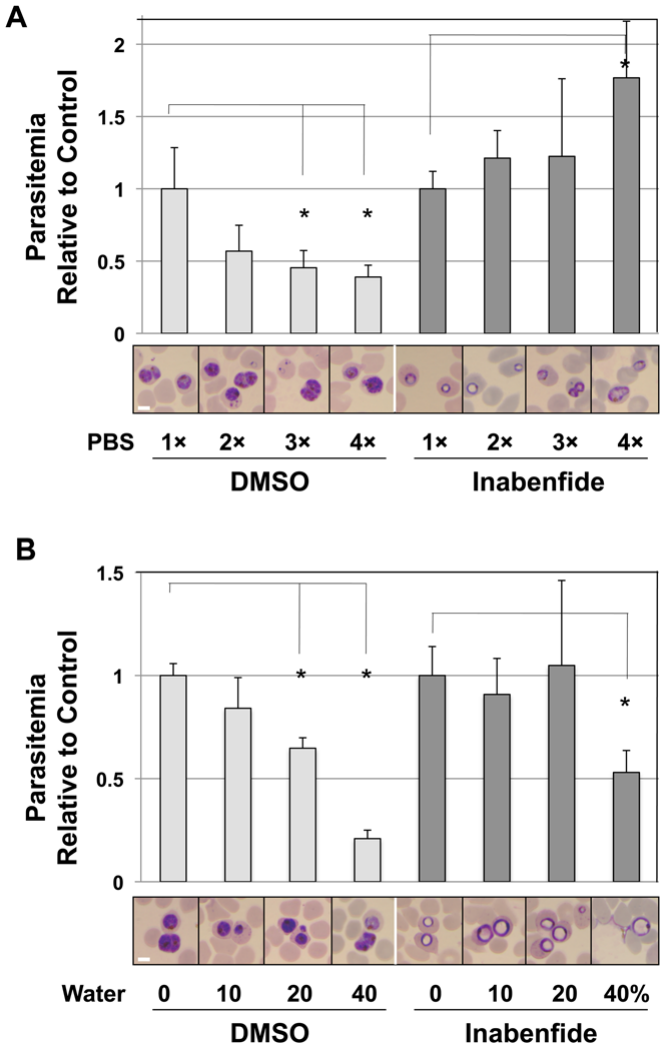
Effects of osmotic pressure to intraerythrocytic parasites treated with INA. (A) Effects of hyperosmotic stress in INA-treated parasites. *P. falciparum* cultures treated with 50 µM INA were observed in various dilution ratios of PBS for 8 h. Parasitemia was determined by Giemsa-stained thin blood smears. Student's *t*-test: *P>0.005. Values are means ± SD of n = 6 in two independent experiments. Data were normalized relative to control cultures (1× PBS) in DMSO- and INA-treated samples, respectively. Representative parasite morphologies are shown for each treatment. Scale bar, 3 µm. (B) Effects of hyposmotic stress, experimentally induced by the addition of water to the culture medium of *P. falciparum*, after 8 h of 50 µM INA treatment. N = 6 smears each in two independent experiments.

Various stressful conditions were applied to INA treated parasite: use of hydraulic pressure, ultrasound, pH (5–9) and temperature changes (4–37°C); but no differences were observed when compared to control parasites (DMSO-treated). However, different responses were seen when trophozoite-stage parasites were under various osmotic stress. Hyperosmotic conditions were achieved by incubating with 10% (v/v) 1×, 2×, 3× or 4× PBS. For control, untreated parasite cultures, relative parasitemia drastically decreased with increasing hyperosmotic environment (Fig. 5 A). However, relative parasitemia in the INA-treated samples increased in higher hyperosmotic environment, especially in cultures treated with 10% 4× PBS. Moreover, ‘haloed’ trophozoites were less evident in cultures incubated with 3× or 4× PBS. To examine the potential effects of cation species, we made a solution where the net solute was the same to PBS but the concentrations of Na^+^ and K^+^ were reversed (137 mM KCl, 8.1 mM K_2_HPO_4_, 2.68 mM NaCl, and 1.47 mM NaH_2_PO_4_). Between normal and reversed PBS in the different treatments, no significant differences were observed in both parasitemia and morphology (Fig. S6). Exogenously added Ca^2+^ (100 µM) to cultures did not help reverse the ‘haloed’ effects of INA in trophozoites (Fig. S7).

Hyposmotic stress enhanced INA's effect in treated parasites: early trophozoite-stage parasites swelled even more by the addition of 10% (v/v) water to the culture medium (Fig. 5 B). At 40% (v/v) water, trophozoites swelled to almost the same volume of the infected erythrocyte. Decrease in parasitemia was also statistically significant compared to the control. These observations suggest that INA treatment might affect membrane permeability; water or ion influx might cause swelling of the parasites.

### Isoprenoid biosynthetic pathway in apicomplexan parasites

Parasite cultures were incubated with gibberellin A_3_ and A_4_, the most effective gibberellins, with or without gibberellin biosynthetic inhibitors. Of note, gibberellins did not affect growth of parasites nor overcome the effects of the inhibitors (data not shown), suggesting that the inhibitors could not disrupt gibberellin biosynthesis in *P. falciparum* or malaria parasites do not utilize gibberellins.

**Figure 6:**
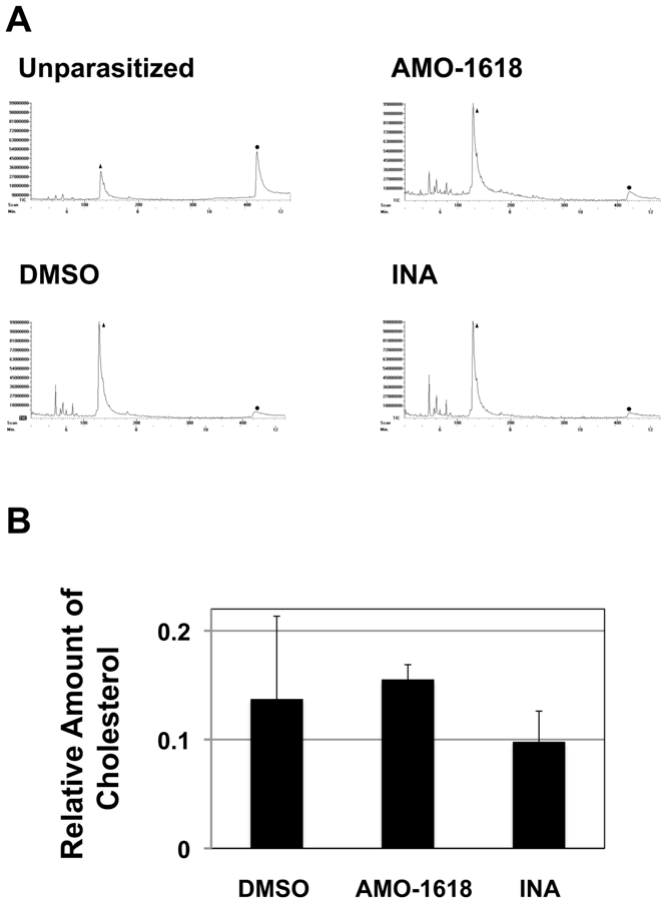
GC-MS analysis of isoprenoids in *P. falciparum in vitro* cultures. (A) Total ion chromatograms of extracts from unparasitized erythrocytes (Unparasitized), parasites treated with 0.1% DMSO, 250 µM AMO-1618, and 50 µM INA for 6 h. Triangles and circles indicate the peaks of geranylgeraniol mixed as an internal control (retention time = 6.57 min) and cholesterol (11.23 min), respectively. These compounds were identified by direct comparison with authentic samples. (B) Quantification of cholesterol in each treated sample. Cholesterol amount was calculated from the peak areas and normalized relative to the ratio of the internal control geranylgeraniol. Values are means and SD of triplicate measurements of a representative experiment.

Gibberellins are diterpene (isoprenoid), ubiquitous and essential compounds by themselves or as materials of secondary metabolites and signal molecules in animals, plant and bacteria [15]. Biosynthetic pathways of isoprenoids are very complicated and parallel pathways for the biosynthesis of the universal 5-carbon building block for all terpenoid compounds exist. A huge variety of sterols and terpenoids are synthesized via isoprenoid metabolic pathways in a species-specific manner, although all pathways utilize farnesyl pyrophosphate as a starting molecule generated in the mevalonate pathway and the 2-*C*-methyl-D-erythritol 4-phosphate (MEP) pathway (Fig. 1) [23]. Gibberellin biosynthetic inhibitors have been known to block other enzymes leading to the production of sterols and terpenoids. We then analyzed *P. falciparum* treated with INA and AMO-1618 by GC-MS. Interestingly, any sterols or terpenoids besides cholesterol derived from human erythrocytes were not detected, and there were no significant differences in the level of cholesterol between control and INA- or AMO-1618-treated samples (Fig. 6).

## Discussion

Efforts on malaria control have been complicated by emergence of drug resistance. With the apicoplast considered to originate from an ancestor with an endosymbiotic alga, and that many nonphotosynthetic processes are shared peculiarly with plants, plant growth regulators are of particular interest for pharmaceutical leads on the assumption that the molecular target may not be present in humans. The present study clearly showed some inhibitors that antagonize gibberellin biosynthesis in plants are effective to both *P. falciparum* and *T. gondii* (Table 1). Gibberellin is one of 5 major plant hormones known to exert various effects on physiological events, particularly germination and seed bearing by promoting cell elongation [24]. 126 different gibberellins are known to occur in plants [15], and they are synthesized via the mevalonate pathway and the MEP pathway. Interestingly, gibberellin has been utilized to induce development of parthenocarpous fruits in some fruits such as grapes. On the other hand, gibberellin inhibitors have also been widely used for agriculture, *e.g.* for dwarf plants without harmful effects to either plants or animals.

All gibberellin inhibitors tested in this study block biosynthesis of gibberellin in plants: AMO-1618 and FC-907 block synthesis of *ent*-kaurene (an intermediate during the synthesis of gibberellin) by inhibition of copalyl-diphosphate synthase (CPPS) and *ent*-kaurene synthase (KS, Fig. 1) [15]. Next to AMO-1618 and FC-907 steps, INA, paclobutrazol, uniconazole P and ancymidol interrupt *ent*-kaurene oxidase (KO), a member of the cytochrome P450 monooxygenases (CYP) oxidating *ent*-kaurene into *ent*-kaurenoic acid. Azole antifungal such as fluconazole and diniconazole are analogous compounds to these inhibitors and interrupt synthesis of ergosterol [25]. They have an asymmetric carbon and their enantiomers are known to differ significantly in their biological properties [16], [26]. (*R*)-forms demonstrate stronger activity than (*S*)-forms in terms of fungicidal effects, whereas the latter is functionally active and the (*R*)-forms showed only residual activity with regard to blockage in plant gibberellin biosynthesis. We tested the stereochemical selectivity of INA and found that the (*S*)-form inhibited growth of *P. falciparum* >2 times stronger than (*R*)-form but *vice versa* in *T. gondii* (Table 1). The stereochemical discrimination was, thus, similar to plants than to fungi in *P. falciparum*, although the (*S*)-form was reportedly 1000 times more potent than (*R*)-form in growth retardation of pumpkin seedlings [26]. Differences may be due to discrepancies in protein structures that relate to membrane permeability or translocation of the enantiomers to the site of biosynthesis in such distant species.

The observed decrease in parasitemia in INA-treated cultures correlated with remarkable morphological changes: swelling and rupture of *P. falciparum* cells, particularly when drug treatment commenced during early trophozoite stage (Fig. 2). Trophozoite-stage parasite cells appear to be ‘haloed’, the center of the cell remains unstained by Giemsa and acridine orange. Both nuclei and cytoplasm were seen on the periphery of the parasite cells. These data suggest that some substances that are neither acidic nor alkaline might accumulate in the unstained parts.

We also observed the intracellular structures in INA-treated cultures using different dyes and found that various intracellular organelles, especially organelles synthesized during schizogony (such as mitochondria, membrane network and food vacuoles) are morphologically abnormal or compromised after short treatment with INA (Fig. 3). Membrane potential in the mitochondria was reduced rapidly within 2 h of INA treatment. Organelle membranes such as those in the ER and nuclear envelope appeared thicker and gaps were observed around such organelles under electron microscopy (Fig. 4). From these observations, it appears that gibberellin biosynthetic inhibitors damage the parasite internal organelles, compromises parasite membranes and results to abnormalities in the treated parasites.

In order to further probe for clues that can shed light into the mechanisms for such changes in the membranes, we exposed INA-treated parasite cells to various stresses, and found that osmotic pressure modified the effects of INA. Treated parasite cells under hyperosmotic conditions became less swelled and viability was significantly restored (Fig. 5 A). In contrast, the addition of water in the culture medium enhanced expansion and rupture of parasites (Fig. 5 B). These results demonstrate that INA treatment might affect permeability of membranes, and consequently result to uncontrollable influx of water or ion into parasite cells. No abnormalities in the plasma membrane structure were observed by electron microscopy (Fig. 4), although this cannot be completely ruled out since parasites with severely compromised plasma membranes are very likely susceptible to rupture or bursting during various treatments for microscopy.

In order to maintain the various functions and stability of plasma membranes, different organisms synthesize several specific components for their biological membranes. For example, sterols, a vast family of isoprenoids, take pivotal role with mammalian and fungal cells generally synthesizing one major sterol, cholesterol and ergosterol, respectively [23], [27]. Plants, on the other hand, are known to produce a characteristically complex sterol mixture. As many as 61 sterols and isoprenoids have been identified in a single maize seedling [28]. Prokaryotes produce hopanoids, pentacyclic isoprenoids, by direct cyclization of squalene (Fig. 1) [29]. The functions of the hopanoids were shown to be equivalent to sterols: functioning as membrane reinforcers affecting membrane permeability and fluidity. In protozoa, there has been one report that *Tetrahymena* synthesizes tetrahymanol, a quasi-hopanoid, together with small amounts of diplopterol [30], [31]. Both components were incorporated into its membranes.

Enzymes that are involved in gibberellin biosynthesis are also of importance in the formation of such sterols and other membrane constituents. Change in the level of these substances after treatment with inhibitors has been often observed as side activities for these inhibitors [15]. For example, AMO-1618 inhibits syntheses of phytosterols and cholesterol, by blocking cycloartenol synthase (CAS) in plants and lanosterol synthase (LS) in animals, respectively [32].

We analyzed *P. falciparum* treated with INA and AMO-1618 by GC-MS in order to identify sterols or isoprenoids in malaria parasites, but we could not detect any isoprenoids, besides cholesterol (Fig. 6). We might be able to hypothesize that responsible components could be too low or structurally unknown to detect by GC-MS in parasite cells. Data about syntheses of isoprenoids in protozoa, especially in *Apicomplexa*, remains nil. Intraerythrocytic parasites are known to intake large amounts of erythrocyte contents with surrounding membranes that are cholesterol rich, however, cholesterol is greatly depleted in parasite membranes (Fig. 6 A) [33]. How parasites transport and dispose of excess cholesterol within the cells remain a puzzle. Additionally, does lack of isoprenoids make membranes leaky even in intracellular parasites? A previous study has demonstrated by differential scanning calorimetry and electron spin resonance the association of gibberellin molecules with phospholipid membranes thereby altering the fluidity or viscosity of the lipid bilayer [34]. Clearly, further studies on gibberellin biosynthetic inhibitors could shed light if these compounds could perturb lipid bilayers or alter properties of the membrane phospholipid.

The gibberellin biosynthetic inhibitors are known to be specific for isoprenoid biosynthesis in plants. We searched amino acid sequences and conserved domain sequences essential for these enzymes using PlasmoDB, but no sequences with significant homologies were found. This may imply the uniqueness of isoprenoid metabolic pathways in *Apicomplexa*, and consequently, provide potent targets to develop novel therapeutic agents.

## Supporting Information

Figure S1 **Concentration-response curve of INA.** Each point represents the mean ± standard deviations (SD) from three independent experiments, with each treatment duplicated twice.
(TIF)

Figure S2 **Chiral separation of enantiomeric INA.** Racemic form of INA was separated on Chiralcel OD column. Mobile phase: n-hexane-2-propanol (8∶2, v/v), flow rate:10 ml/min, detection: 275 nm. HPLC chromatograms of each enantiomer are also shown.
(TIF)

Figure S3 **Effect of gibberellin biosynthetic inhibitors on the intraerythrocytic development of** ***P. falciparum*** **.** Asynchronized parasites were treated with 200 µM FC-907 for 24 h, 500 µM uniconazole P for 8 h, 200 µM paclobutrazol for 8 h, 50 µM INA or 1 µl/ml DMSO for 6 h. Giemsa-stained thin blood smears were prepared from each sample after the indicated treatment and examined under a microscope. Each panel shows the typical morphology of trophozoite-stage parasites in each treatment. Scale bar, 3 µm.
(TIF)

Figure S4 **Effects of gibberellin biosynthetic inhibitors on the viability of erythrocytes to support growth of** ***P. falciparum*** **.** Erythrocytes that had been incubated in RPMI 1640 containing 10% human serum and supplemented with 1 µl/ml DMSO, 50 µM INA or 250 µM AMO-1618 for 24 h were washed with RPMI 1640 twice and mixed with *P. falciparum* infected erythrocytes at 0.3% starting parasitemia. Thin blood films from each culture were prepared after 48 h, stained with Giemsa and parasitemias were counted under a microscope. Values are mean ± SD of n = 3 in each representative experiment.
(TIF)

Figure S5 **Influence of INA concentration on growth inhibition in** ***P. falciparum*** **.** (A) Synchronized parasites at early trophozoite stage were treated with 0 (1 µl/ml DMSO), 10 or 50 µM INA for 0–8 h. Parasitemia was determined by thin blood films after staining with Giemsa. Error bars represent the standard deviation (SD) of three independent experiments made in duplicate. Data are normalized relative to those for the control treated for 0 h. (B) The parasites treated with 0, 10 or 50 µM INA after 4 h, stained with Giemsa, and visualized under light microscope. Scale bar = 3 µm. Each panel shows the typical morphology of trophozoite-stage parasites in each treatment.
(TIF)

Figure S6 **Effects of cation species to the parasitemia of the intraerythrocytic parasites treated with INA.** Synchronized parasites at early trophozoite stage were mixed with 10% of 1× and 4× normal PBS or the solution that Na^+^ and K^+^ concentrations in PBS are exchanged (reversed-PBS; 1× reversed-PBS contains 137 mM KCl, 8.1 mM K_2_HPO_4_, 2.68 mM NaCl, 1.47 mM NaH_2_PO_4_), and treated with 50 µM INA or 1 µl/ml DMSO for 8 h. Parasitemia was determined by counting thin blood film from each culture blindly after staining with Giemsa; the counter was blinded to sample identities. Error bars represent the standard deviation of three independent experiments made in duplicate. Data are normalized relative to those for the control treated with 1× PBS and 1 µl/ml DMSO.
(TIF)

Figure S7 **Influence of Ca^2+^ on the effects of INA in** ***P. falciparum*** **.** Parasites were synchronized by 5% sorbitol treatment and treated with 1 µl/ml DMSO or 50 µM INA with or without adding 100 µM CaCl_2_ after 18 h of incubation. The parasites were examined at 6 h of the treatment by thin blood smears and staining with Giemsa. Scale bar indicates 3 µm. Each panel shows a typical morphology of trophozoite-stage parasites in each treatment.
(TIF)
