# Supplementary figures and images for: Gibberellin Biosynthetic Inhibitors Make Human Malaria Parasite Plasmodium falciparum Cells Swell and Rupture to Death
Source: PLoS One. 2012 Mar 7;7(3):e32246. doi: 10.1371/journal.pone.0032246 (PMC3296703; doi:10.1371/journal.pone.0032246)

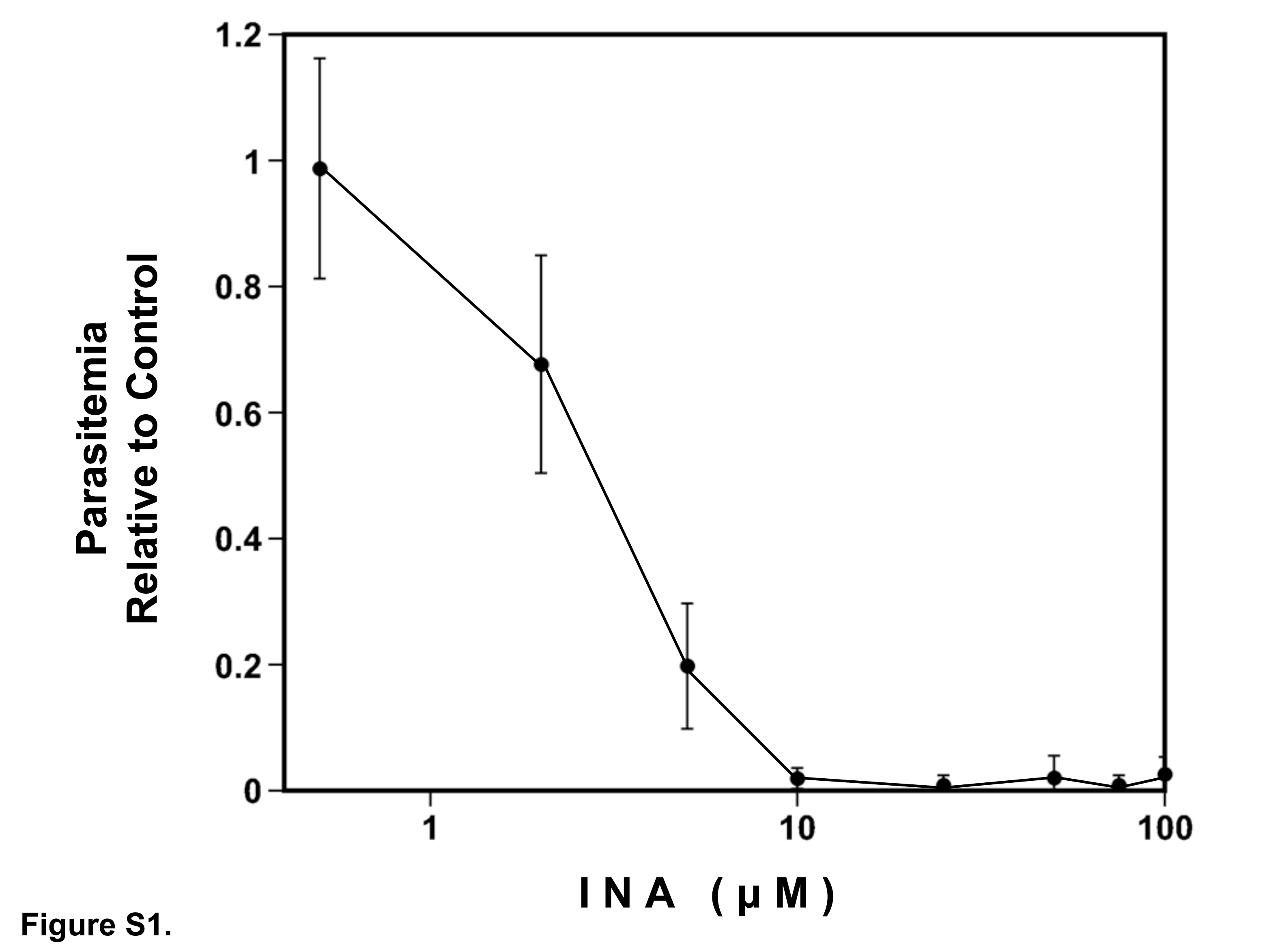

Supplement: Figure S1 — Concentration-response curve of INA. Each point represents the mean ± standard deviations (SD) from three independent experiments, with each treatment duplicated twice. (TIF) [file pone.0032246.s001.tif]

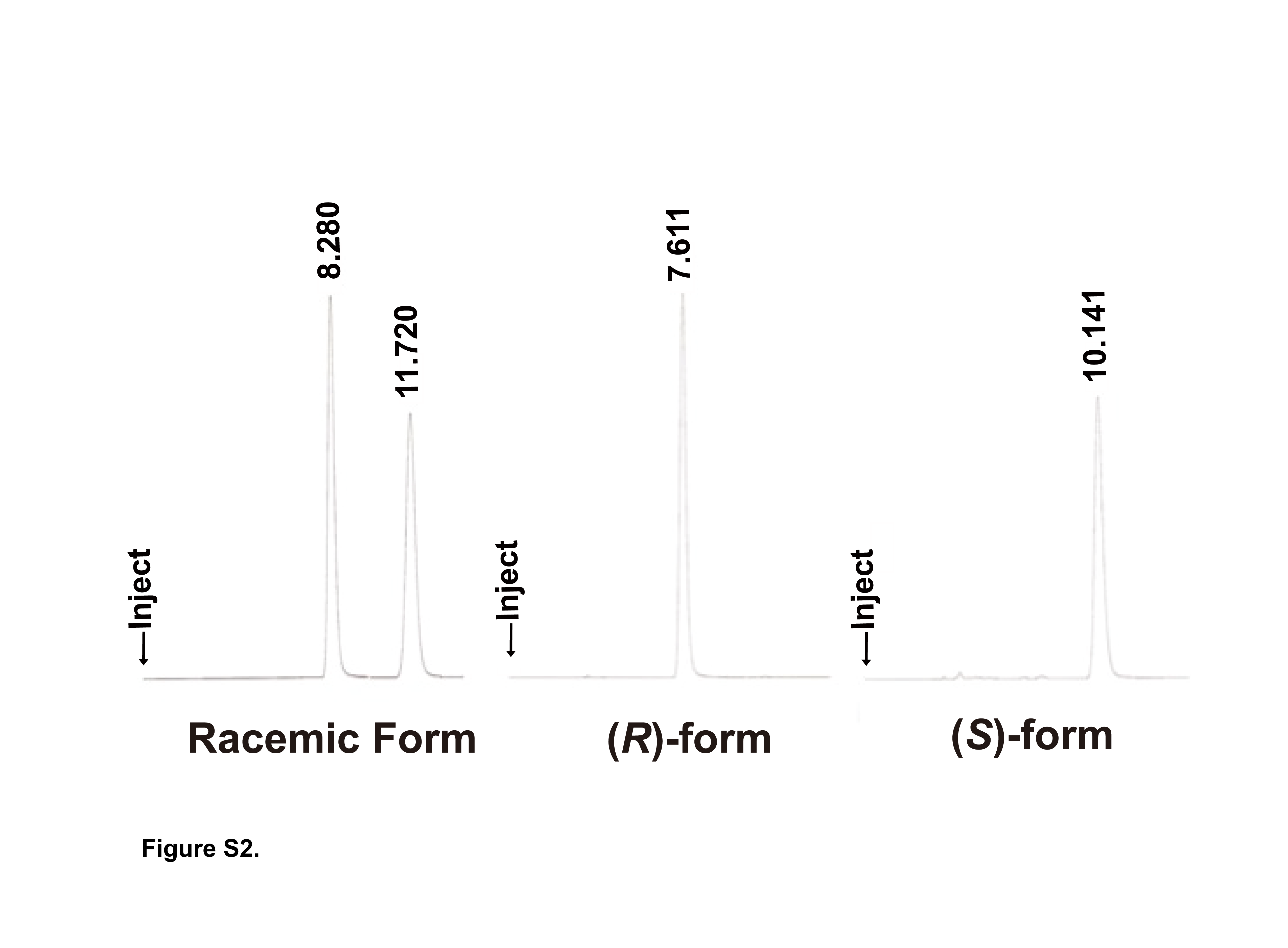

Supplement: Figure S2 — Chiral separation of enantiomeric INA. Racemic form of INA was separated on Chiralcel OD column. Mobile phase: n-hexane-2-propanol (8∶2, v/v), flow rate:10 ml/min, detection: 275 nm. HPLC chromatograms of each enantiomer are also shown. (TIF) [file pone.0032246.s002.tif]

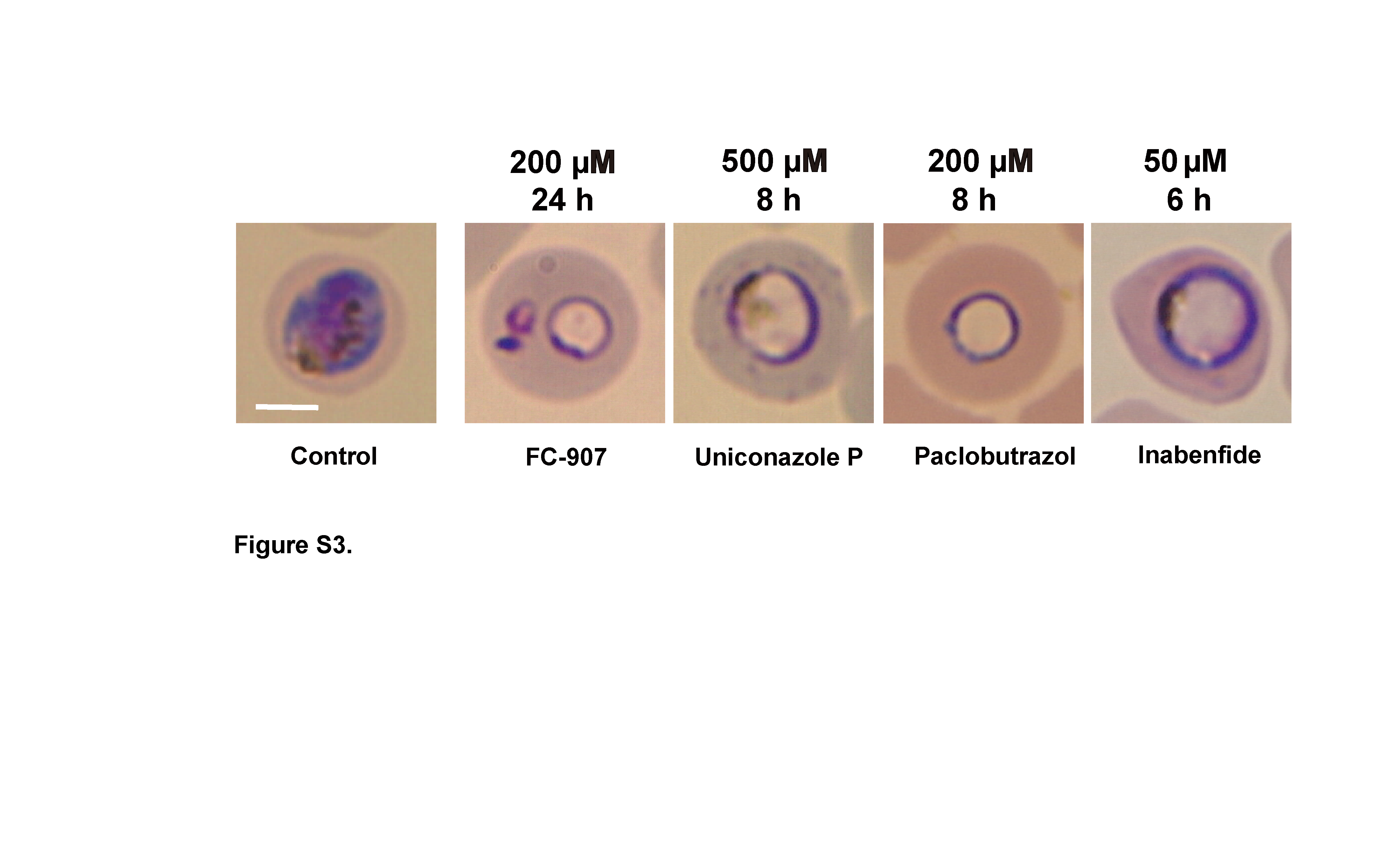

Supplement: Figure S3 — Effect of gibberellin biosynthetic inhibitors on the intraerythrocytic development of P. falciparum . Asynchronized parasites were treated with 200 µM FC-907 for 24 h, 500 µM uniconazole P for 8 h, 200 µM paclobutrazol for 8 h, 50 µM INA or 1 µl/ml DMSO for 6 h. Giemsa-stained thin blood smears were prepared from each sample after the indicated treatment and examined under a microscope. Each panel shows the typical morphology of trophozoite-stage parasites in each treatment. Scale bar, 3 µm. (TIF) [file pone.0032246.s003.tif]

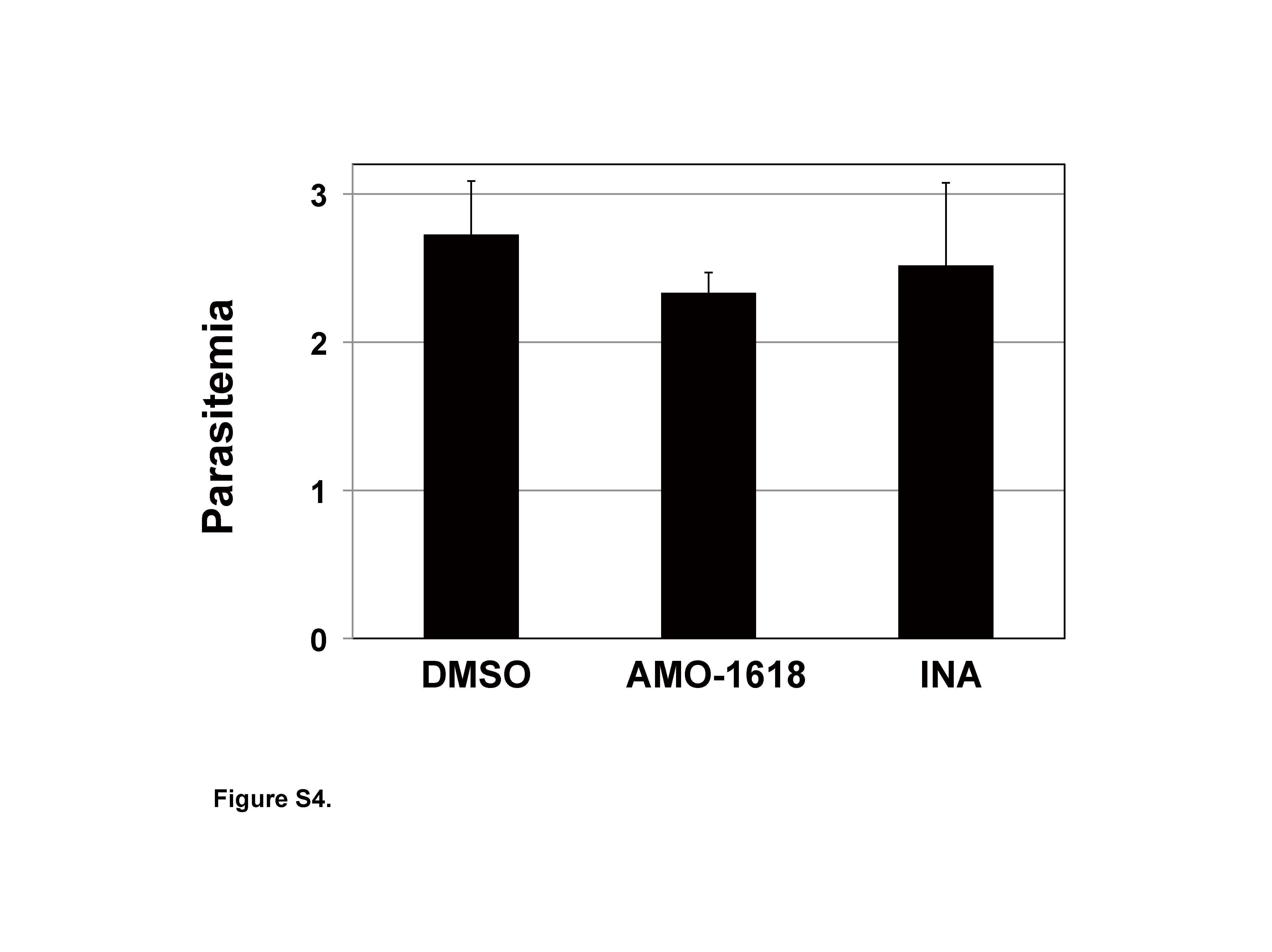

Supplement: Figure S4 — Effects of gibberellin biosynthetic inhibitors on the viability of erythrocytes to support growth of P. falciparum . Erythrocytes that had been incubated in RPMI 1640 containing 10% human serum and supplemented with 1 µl/ml DMSO, 50 µM INA or 250 µM AMO-1618 for 24 h were washed with RPMI 1640 twice and mixed with P. falciparum infected erythrocytes at 0.3% starting parasitemia. Thin blood films from each culture were prepared after 48 h, stained with Giemsa and parasitemias were counted under a microscope. Values are mean ± SD of n = 3 in each representative experiment. (TIF) [file pone.0032246.s004.tif]

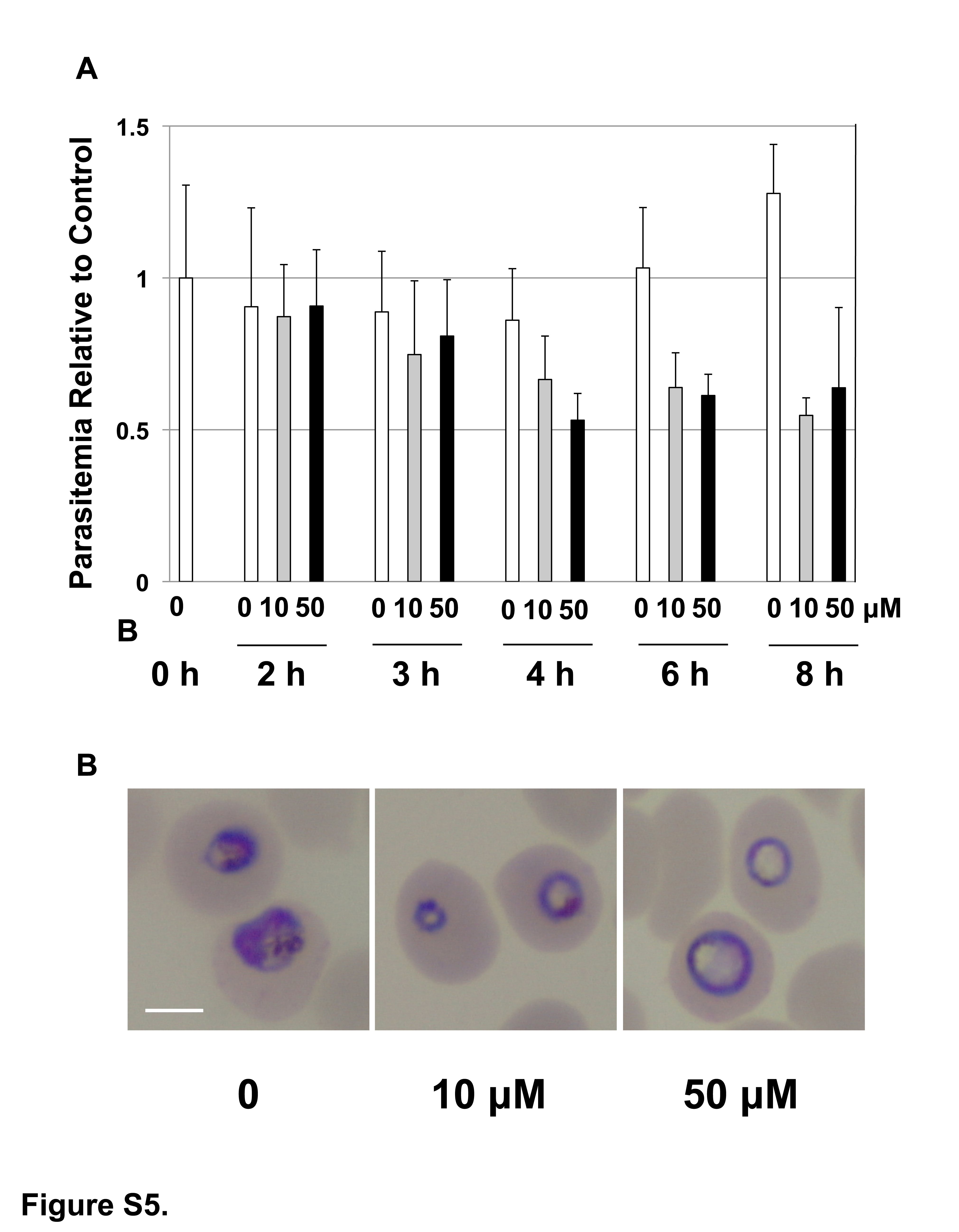

Supplement: Figure S5 — Influence of INA concentration on growth inhibition in P. falciparum . (A) Synchronized parasites at early trophozoite stage were treated with 0 (1 µl/ml DMSO), 10 or 50 µM INA for 0–8 h. Parasitemia was determined by thin blood films after staining with Giemsa. Error bars represent the standard deviation (SD) of three independent experiments made in duplicate. Data are normalized relative to those for the control treated for 0 h. (B) The parasites treated with 0, 10 or 50 µM INA after 4 h, stained with Giemsa, and visualized under light microscope. Scale bar = 3 µm. Each panel shows the typical morphology of trophozoite-stage parasites in each treatment. (TIF) [file pone.0032246.s005.tif]

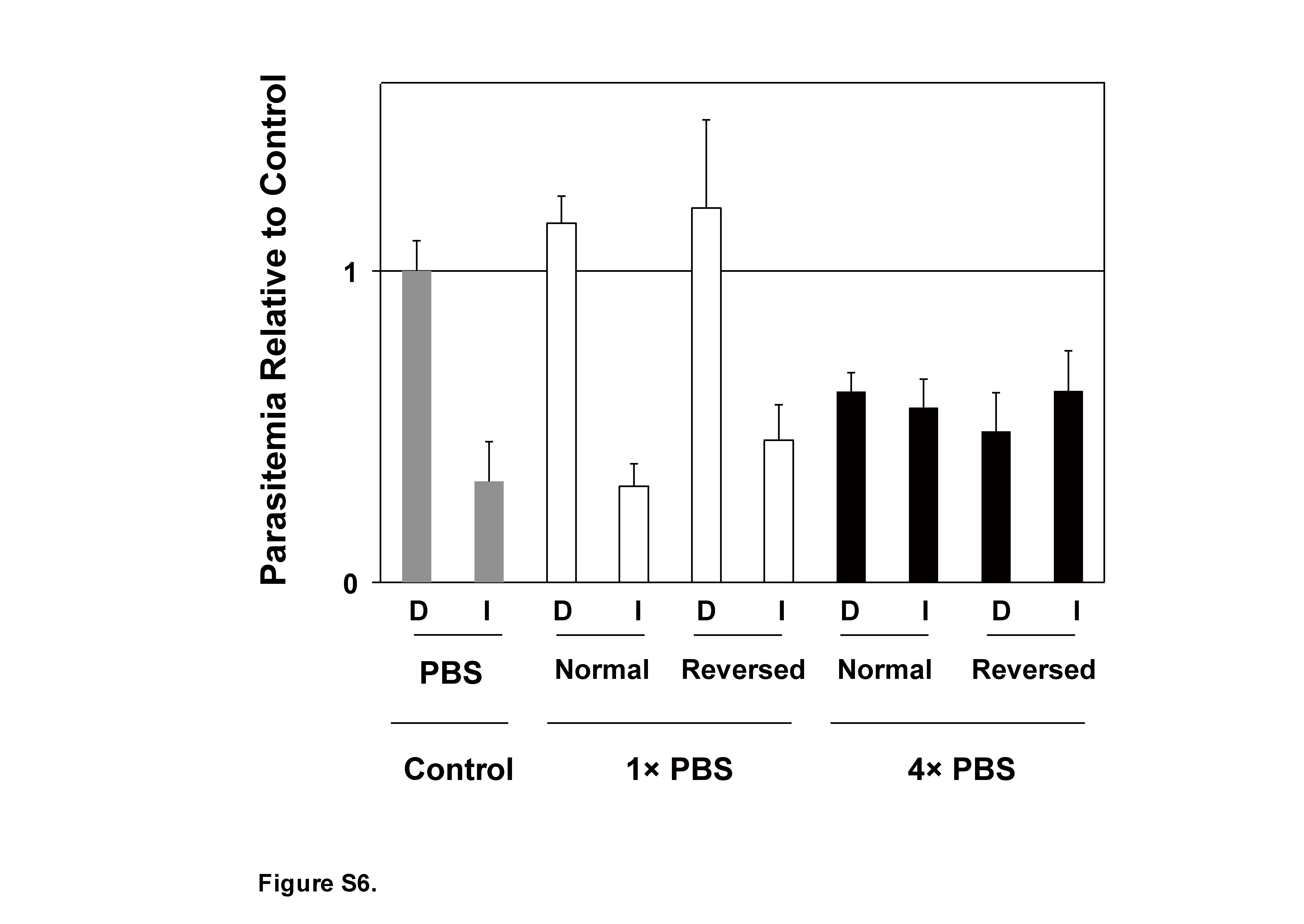

Supplement: Figure S6 — Effects of cation species to the parasitemia of the intraerythrocytic parasites treated with INA. Synchronized parasites at early trophozoite stage were mixed with 10% of 1× and 4× normal PBS or the solution that Na+ and K+ concentrations in PBS are exchanged (reversed-PBS; 1× reversed-PBS contains 137 mM KCl, 8.1 mM K2HPO4, 2.68 mM NaCl, 1.47 mM NaH2PO4), and treated with 50 µM INA or 1 µl/ml DMSO for 8 h. Parasitemia was determined by counting thin blood film from each culture blindly after staining with Giemsa; the counter was blinded to sample identities. Error bars represent the standard deviation of three independent experiments made in duplicate. Data are normalized relative to those for the control treated with 1× PBS and 1 µl/ml DMSO. (TIF) [file pone.0032246.s006.tif]

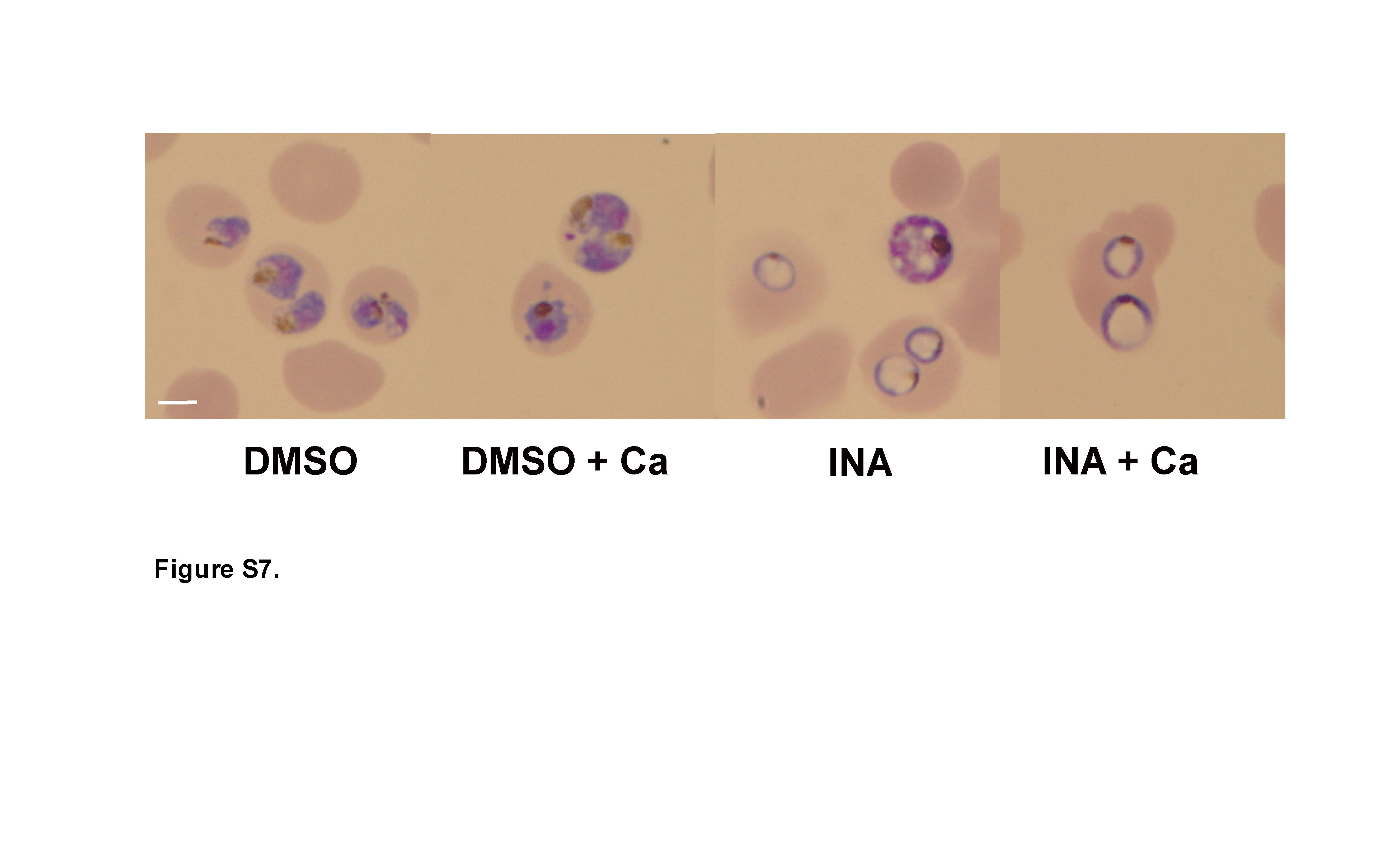

Supplement: Figure S7 — Influence of Ca2+ on the effects of INA in P. falciparum . Parasites were synchronized by 5% sorbitol treatment and treated with 1 µl/ml DMSO or 50 µM INA with or without adding 100 µM CaCl2 after 18 h of incubation. The parasites were examined at 6 h of the treatment by thin blood smears and staining with Giemsa. Scale bar indicates 3 µm. Each panel shows a typical morphology of trophozoite-stage parasites in each treatment. (TIF) [file pone.0032246.s007.tif]
